# Molecular pathogenesis of rhegmatogenous retinal detachment

**DOI:** 10.1038/s41598-020-80005-w

**Published:** 2021-01-13

**Authors:** Tiina Öhman, Lisa Gawriyski, Sini Miettinen, Markku Varjosalo, Sirpa Loukovaara

**Affiliations:** 1 Institute of Biotechnology and Helsinki Institute of Life Science, University of Helsinki, Viikinkaari 1, P.O. Box 65, 00014 Helsinki, Finland; 2 Department of Ophthalmology, Unit of Vitreoretinal Surgery, Helsinki University Hospital, Haartmaninkatu 4 C, 00290 Helsinki, Finland; 3 Individualized Drug Therapy Research Program, Faculty of Medicine, University of Helsinki, Helsinki, Finland

**Keywords:** Retinal diseases, Proteomics, Proteomic analysis

## Abstract

Rhegmatogenous retinal detachment (RRD) is an ophthalmic emergency, which usually requires prompt surgery to prevent further detachment and restore sensory function. Although several individual factors have been suggested, a systems level understanding of molecular pathomechanisms underlying this severe eye disorder is lacking. To address this gap in knowledge we performed the molecular level systems pathology analysis of the vitreous from 127 patients with RRD using state-of-the art quantitative mass spectrometry to identify the individual key proteins, as well as the biochemical pathways contributing to the development of the disease. RRD patients have specific vitreous proteome profiles compared to other diseases such as macular hole, pucker, or proliferative diabetic retinopathy eyes. Our data indicate that various mechanisms, including glycolysis, photoreceptor death, and Wnt and MAPK signaling, are activated during or after the RRD to promote retinal cell survival. In addition, platelet-mediated wound healing processes, cell adhesion molecules reorganization and apoptotic processes were detected during RRD progression or proliferative vitreoretinopathy formation. These findings improve the understanding of RRD pathogenesis, identify novel targets for treatment of this ophthalmic disease, and possibly affect the prognosis of eyes treated or operated upon due to RRD.

## Introduction

Retinal detachment (RD) is an ocular emergency severely damaging the visual function of the patient and often requiring prompt surgery to prevent further advancement of detachment and to restore sensory function^1,2^. In RD the highly organized multilayer neurosensory retina separates from the underlying retinal pigment epithelium (RPE), a single-cell layer of cuboidal polarized cells at the posterior part of the eye. Retinal detachments can be rhegmatogenous (caused by a break in the retina) or non-rhegmatogenous [due to the accumulation of subretinal fluid under the retina (exudative retinal detachment) or scar tissue pulling on the retina (tractional retinal detachment)] or a combination of these. Rhegmatogenous retinal detachment (RRD) is the most common type of retinal detachment, with an annual incidence rate of ca. 1/10,000 persons. It has an increased chance of occurring in patients with increased age, myopia, previous cataract surgery or trauma^3^.

The connection between the RPE and photoreceptor cells is essential to the health of photoreceptor cells^4^. After retinal detachment, nutrition to the photoreceptor cells is impaired and the photoreceptor cells begin to degenerate rapidly (within hours or few days) via apoptosis and programmed cell death^5^. The RPE cells and fibroblasts will then abnormally and ectopically proliferate, thereby resulting in irreversible damage to visual function. In addition to structural changes in the retina, complex biomolecular mechanisms activated after RRD may also play an important role in its pathogenesis. In fact, numerous cytokines, pro-inflammatory and growth factors are released to vitreous during RRD^6^. It has been suggested that these molecules play a significant role in the injury-induced wound-healing process and apoptosis of retinal photoreceptors in RRD^7,8^.

As a complication, a prolonged RRD can develop to a proliferative vitreoretinopathy (PVR)—the most advanced RRD disease stage. PVR complicates 5–11% of RRD eyes, leading ultimately to the irreversible loss of sight of the affected eye^8,9^. Structurally, the PVR process manifests as neuroretinal star-fold scar tissue that wrinkles the retinal architecture and is associated with preretinal, intraretinal, and/or subretinal fibrotic membranes. Despite numerous efforts, no effective preventive or pharmacological treatment strategies are available to reduce the risk of PVR fibrosis development in eyes with RRD^10,11^.

Quantitative proteomics has become an indispensable method of providing molecular level information on the pathogenesis, diagnosis, and treatment of ophthalmic diseases, primarily focusing on the acellular vitreous, where protein content reflects well with the pathogenesis of the surrounding tissues^12^. Currently, the majority of proteomic studies characterizing disease‐induced vitreous proteome changes have been analyzed using data-dependent acquisition (DDA) method where selected number of peptides are sequentially selected for MS2 analysis^13–17^. However, the stochastic precursor selection of DDA can lead to inconsistent detection of peptides and the corresponding proteins in the sample cohort. In this current study, a data‐independent acquisition (DIA) method using SWATH-MS proteomics technique was utilized to find proteins enriched particularly in RRD vitreous. SWATH‐MS is an emerging mass spectrometric method that offers a high degree of quantitative accuracy, proteomic coverage, reproducibility of proteome coverage, and sample throughput^18^.

We therefore performed molecular pathology analysis of the vitreous proteomes collected from 127 patients with RRD using SWATH-mass spectrometry. The aim of the present study was to outline the main molecules and cellular processes activated during RRD and to pinpoint potential novel targets for therapeutic use. Our data indicates unique exosome-mediated proteomic changes in RRD that have not been observed in age-related vitreoretinal interface diseases or proliferative diabetic retinopathy (PDR). In addition, our results provide a view on the role of photoreceptor death, glycolysis, and immune response as well as Wnt and MAPK signaling in the progression of RRD. A deeper understanding of the pathological changes of the entire spectrum of the clinical RRD phase could allow more effective use of existing therapeutic strategies or develop new therapies to improve the visual prognosis of RRD patients.

## Results

### Patients

**Figure 1:**
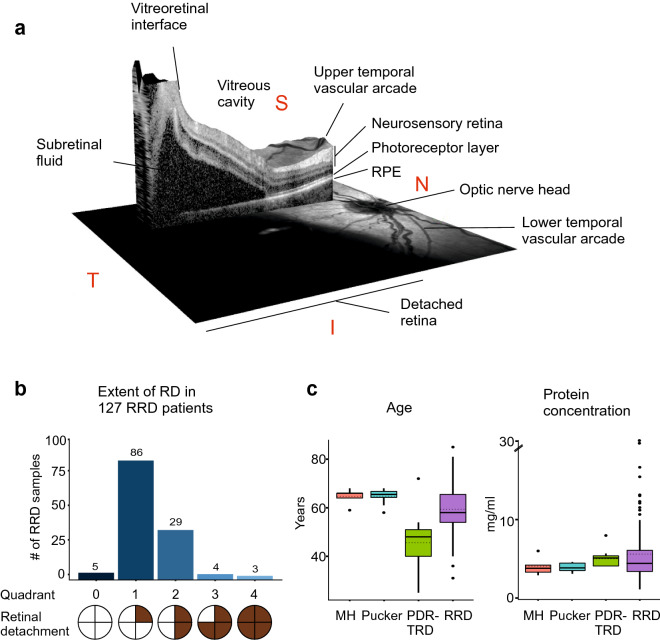
RRD and patient characterization. (**a**) An optical coherence tomography scan of the right eye with acute-onset RRD of a 53-year old female patient reveals fovea-involving RRD (macula off) with edema in the outer retinal layers (right eye; central retinal thickness 594 µm at the fovea). Scale bar: 200 µm. *RPE *retinal pigment epithelium, *T *temporal, *I *inferior, *N *nasal, *S *superior. (**b**) Distribution of detached retinal area in studied RRD patients. Quadrant 0 = a large tear/rupture with starting (early-onset) local retinal detachment (RD), quadrant 1 = 25% of RD (covering one quadrant of retinal area), 2 quadrants = 50% of RD, 3 quadrants = 75% of RD and 4 quadrants = a total retinal detachment. (**c**) The demographics of the MH-Pucker, PDR-TRD and RRD patients, showing the distributions of the age (years) and vitreous protein concentration (mg/ml). The median is shown with a line and the mean with a dashed line.

This study consisted of vitreous samples from 127 RRD patients (66 male and 61 female). A 3D-image obtained with OCT from the right eye of a representative RRD-patient shows a large fovea involving retinal detachment (Fig. 1a). RRD eyes used in this study were categorized using quadrants according to the extent of detachment at the time of primary vitrectomy. Five patients had a large retinal tear/rupture with early-onset local retinal detachment (RD) (quadrant 0), 86 patients had a 25% RD (covering at most one quadrant of retinal area), 29 patients had a 50% RD (2 quadrants), 4 patients had 75% RD (3 quadrants), and 3 patients had a total detachment (4 quadrants) (Fig. 1b). Majority of the patients in this study had acute-onset RRD (less than 2 quadrants), operated within the first few days after development of RRD. PVR was observed in a total of eight eyes. For comparison, samples of neurodegenerative vitreoretinal interface eyes and proliferative diabetic retinopathy eyes with tractional-retinal detachment (PDR-TRD) were used as a control^15,16^. Age distributions of the patients were 59.5 ± 10.1 in RRD group, 64.6 ± 3.4 in MH group, 64.8 ± 3.2 in Pucker group and 45.7 ± 14.2 in PDR-TRD group, showing that the MH and Pucker patients were slightly older and diabetic patients slightly younger than RRD patients (Fig. 1c). The average protein concentration did not vary significantly between the sample groups (Fig. 1c). However, two outliers with very high protein content were observed in the RRD samples compared to the other RRD-samples. These eyes belong to patients with the most advanced RRD (4 quadrants with PVR), having protein concentration 22.1 and 27.1 mg/ml. This observation was in line with a previous report, showing that there is more protein in the anterior chamber of the eye if the RRD has lasted longer and the area of retinal detachment is larger^19^. Detailed clinical characteristics are given in Table 1.

**Table 1:** Clinical information of the patients.

Ophthalmic disease	RRD	MH	Pucker	PDR-TRD
No. of patients	127	5	10	9
Age (mean ± SD)	59.5 ± 10.1	64.6 ± 3.4	64.8 ± 3.2	45.7 ± 14.2
Sex (male/female)	66/61	4/1	5/5	5/4
Protein concentration (mg/ml ± SD)	5.5 ± 3.7	4.0 ± 1.2	4.0 ± 0.6	5.0 ± 1.3
BMI (kg/m^2^ ± SD)*	26.6 ± 5.0	22.9 ± 1.9	25.2 ± 4.0	*nd*
Statin (yes/no)*	4/52	1/4	2/8	3/6
Smoking (yes/no)*	7/49	1/4	0/10	*nd*
**Extent of RD**				
Quadrant 0	5	–	–	–
Quadrant 1	86	–	–	–
Quadrant 2	29	–	–	–
Quadrant 3	4	–	–	–
Quadrant 4	3	–	–	–
PVR (yes/no)	8/119	–	–	-

*RRD *rhegmatogenous retinal detachment, *MH* macular hole, *PDR-TRD* proliferative diabetic retinopathy with tractional retinal detachment, *SD* standard deviation, *BMI* body mass index, *RD* retinal detachment, *PVR* proliferative vitreoretinopathy, *nd* no data. *71 RRD patients do not have data.

### SWATH-MS-based quantitative analysis of the RRD vitreous samples

**Figure 2:**
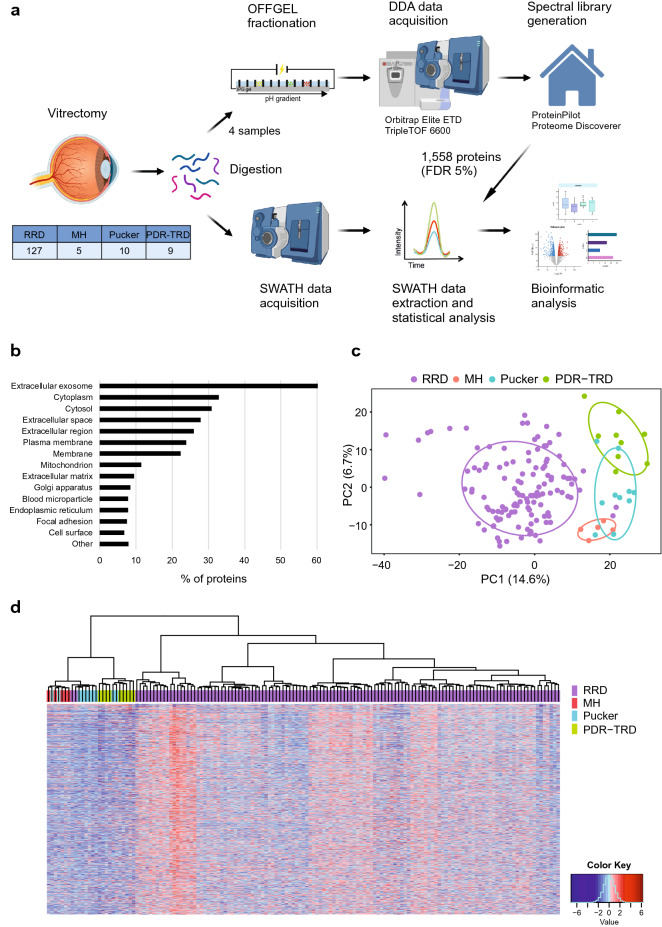
SWATH-MS analysis of vitreous samples. (**a**) Vitreous samples were collected via vitrectomy and proteins were digested with trypsin. For spectral library generation, the resulting peptides were fractionated and analyzed using DDA mode. The spectral library built was then used to extract the peptide and the quantification information of the SWATH runs. Bioinformatics approaches were used to understand biological relevance of the differentially expressed proteins. (**b**) Cellular localization of the detected vitreal proteins were predicted using GO-Cellular Component terms with the DAVID bioinformatics tool. (**c**) Principal component analysis (PCA) and (**d**) hierarchical clustering analysis of the samples showed a clear separation of RRD and control posterior segment eye diseases (MH, Pucker and PDR-TRD). In the heat map all 1177 quantified proteins expression levels are shown as raw Z-score of the SWATH peak area of each sample.

To understand the precise pathophysiological mechanisms of RRD, we performed a global proteome profiling of human vitreous humor samples from 127 RRD patients using label-free quantitative SWATH-MS proteomic analysis. In addition, vitreous samples from 24 individuals with characterized diseases MH, Pucker, or PDR-TRD were analyzed as a control set. The workflow used for SWATH-MS analysis of vitreous samples is shown in Fig. 2a. The SWATH-MS data analysis relies on a reference assay library, which must be sample-specific and of sufficient compositional depth to enable extensive peptide identification. Therefore, a vitreous humor specific reference spectral library was first generated using a DDA analysis of the four fractionated vitreous samples, covering RRD, MH, Pucker, and PDR with TRD patient samples. This resulted in a spectral library consisting of 1 558 human proteins with 5% FDR on the protein level.

In the SWATH-MS experiments, a total of 1 177 proteins were identified and quantified across all 151 vitreous samples (Supplementary Table S1). These vitreous proteins were involved in 78 significant biological processes (adjusted p-value < 0.01), in which ‘proteolysis’ (88 proteins), ‘cell-adhesion’ (80 proteins), ‘oxidation–reduction’ (63 proteins), ‘platelet degranulation’ (58 proteins) and ‘innate immune response’ (53 proteins) were the most abundant (Supplementary Table S2). Interestingly, we also detected many proteins linked to signaling pathways such as the mitogen activated protein kinases (MAPK) cascade, the Wnt signaling, and receptor signaling pathways. When analyzed for cellular components, the identified proteins were primarily categorized as extracellular exosomes (708 proteins; 60%), extracellular space (327; 28%) or plasma membrane (281; 24%) (Fig. 2b). This suggested that most of the vitreous proteins are actively secreted or released from the cell membrane of the surrounding cells by vitreous proteolytic release. In addition, considerable proportion of identified proteins were intracellular and localized either to cytosol (363; 31%) or to the membrane-bound organelle like mitochondria (135; 11%), endoplasmic reticulum (ER, 91; 7%)) or Golgi (99; 8%), indicating cell death or degeneration during RRD.

### Vitreous protein profiles differ between vitreoretinal eye diseases

We performed principal component analysis (PCA) and hierarchical clustering of all quantified proteins from the patient’ vitreous samples. With the exception of a few outlier samples, PCA analysis demonstrated a clear separation between RRD, MH, Pucker and PDR-TRD sample groups (Fig. 2c). A similar result was obtained using hierarchical clustering, in which RRD samples clustered separately from MH, Pucker and PDR-TRD samples (Fig. 2d). As shown previously^16^, MH and Pucker have marked similarities in their protein profiles, and were therefore combined in this and further analyses.

**Figure 3:**
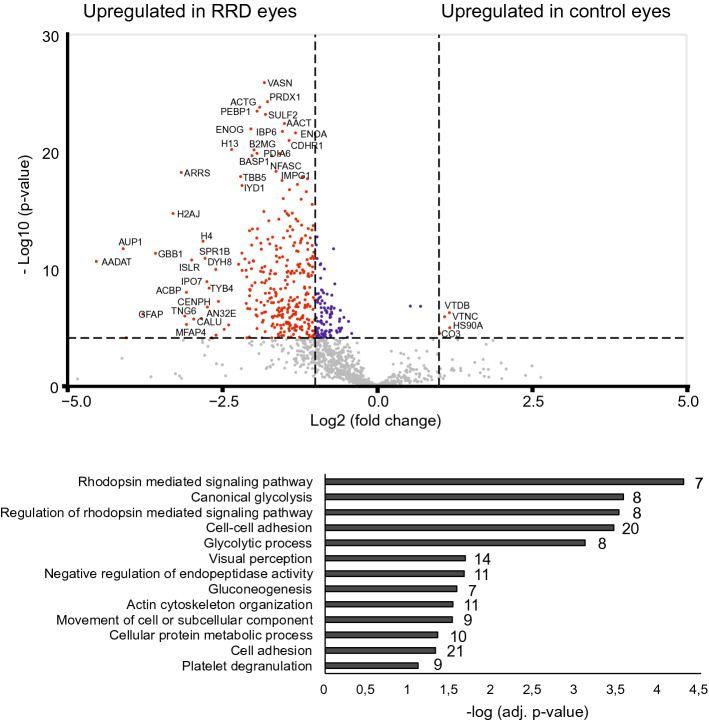
Comparison between RRD and control posterior segment eye disease proteomes. The volcano plots of differentially expressed proteins between RRD and control MH-Pucker and PDR-TRD eye groups. P-value was set to match q-value < 0.1. The red dots indicate significantly differed proteins with q < 0.1 and FC > 2. Significantly upregulated proteins in the RRD samples were categorized by their biological processes.

Since we detected clear differences in RRD proteome compared to MH, Pucker or PDR-TRD proteomes, we next performed Welch's T-test analysis to find the proteins that are present in significantly differential concentrations particularly in the vitreous of RRD patients. When the RRD samples were compared to control samples (n = 24), we detected a difference in the abundances of 406 proteins (q‐value < 0.1) with 313 proteins being more abundant in the RRD samples (abundance ratio > 2) and only 4 proteins more abundant in the control samples (Fig. 3; Supplementary Table S3). The very low number of up-regulated proteins in control samples may be due to a smaller set of samples versus to the RRD group and the biological variation of samples. Because the control sample group was a combination of three diseases, we made additional comparisons with only MH-Pucker samples (n = 15) or only PDR-TRD samples (n = 9) to obtain more detailed information on RRD proteome changes (Supplementary Fig. S1; Supplementary Table S3).

### The significant biological processes in RRD

To get an understanding of the biological processes and pathways active or activated in the RRD, the enriched proteins in the RRD samples were categorized according to their involvement in different biological processes and KEGG pathways (Fig. 3, Supplementary Tables S4 and S5). Rhodopsin mediated signaling pathways and glycolytic processes were the most significant processes detected in RRD, making these the most potential RRD specific events. Additionally, proteins involved in cell adhesion or platelet degranulation were enriched in the RRD proteome, confirming the inflammatory process in the RRD disease. Additionally, several proteasome-mediated signaling pathways were detected especially in MH-Pucker comparison.

**Figure 4:**
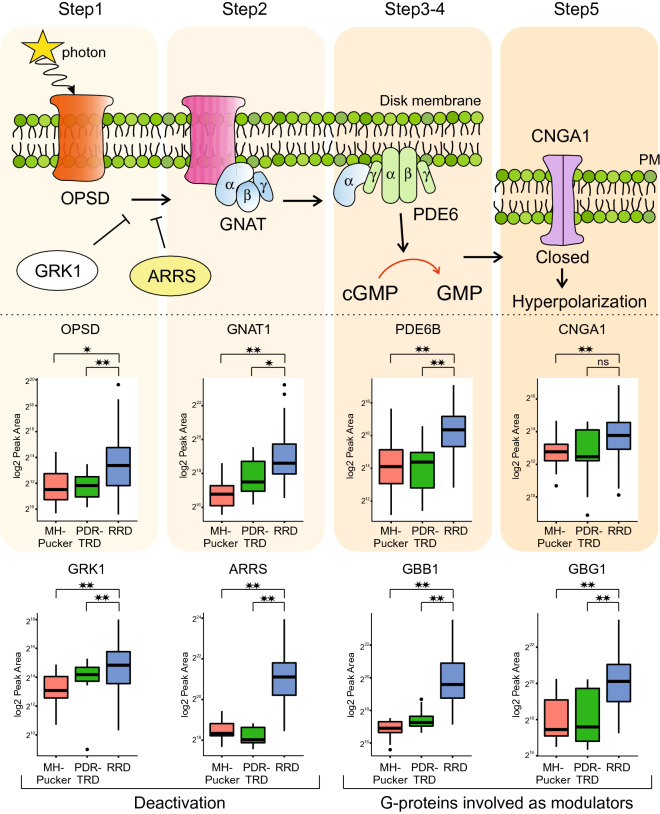
Rhodopsin signaling. Representation of molecular steps in photoactivation (modified from Leskov et al. 2000). Step 1, A light photon activates rhodopsin (OPSD) by conformational change in the disk membrane. Step 2, activated rhodopsin makes contacts with G protein known as transducin (GNAT). Step 3, transducin dissociates from GDP and binds GTP, then the alpha subunit-GTP complex of transducin dissociates from the beta and gamma subunits. Step 4, the alpha subunit of transducin activates phosphodiesterase, also known as PDE6, by binding to one of two regulatory subunits of PDE and inhibits its activity. PDE6 hydrolyzes cGMP, forming GMP. Step 5, the intracellular concentration of cGMP decreases and therefore the CNGA1 cation channels close. Closure of the cation channels causes hyperpolarization of the cell due to the ongoing efflux of potassium ions. Abundance of the 8 proteins linked to rhodopsin signaling upregulated in RRD are shown as boxplots. **q < 0.1; *q < 0.5, *ns *non-significant differences.

The rhodopsin mediated signaling pathway, which is an essential part of phototransduction, was the most significantly enriched process in the RRD. In our analysis, we detected 8 proteins (OPSD, GNAT1, PDE6B, CNGA1, GRK1, ARRS, GBB1 and GBG1) directly linked to rhodopsin signaling (Fig. 4). The phototransduction is a biochemical process by which the photoreceptor cells generate electrical signals in response to captured photons. The signal cascade starts with the absorption of photons by the photoreceptive pigments, the rhodopsins (OPSD), which undergoes a conformational change that activates the rhodopsin. This catalyzes the replacement of GDP by GTP on the heterotrimeric G protein, transducin (GNAT), which in turn, promotes cGMP hydrolysis by cGMP-phosphodiesterase (PDE6), leading to hyperpolarization of the photoreceptor cells. GBB1 and GBG1 are G-proteins involved as a transducer required for the GTPase activity. The hyperpolarization of the membrane potential of the photoreceptor cell modulates the release of neurotransmitters to downstream cells. CNGA1 is the subunit of the rod cyclic GMP-gated cation channel, which is involved in the final stage of the phototransduction pathway. Rhodopsin is deactivated rapidly after activating transducin by rhodopsin kinase (GRK1) and arrestin (ARRS)^20^. Interestingly, the detected proteins were from all activation steps, and also deactivating proteins GRK1 and ARRS, and G-proteins GBB1 and GBG1 involved in signaling pathway regulator, were detected. In addition, we observed a clear enrichment of other photoreceptor proteins, classified by a term ‘*visual perception’*. These include e.g. interphotoreceptor matrix proteoglycan 1 and 2 (IMPG1 and IMPG2) which interact with hyaluronan and act as a structural constituent of interphotoreceptor matrix, and also peripherin-2 and rod outer segment membrane protein 1 (ROM1), which play a role in the rod outer segment (ROS) morphogenesis.

**Figure 5:**
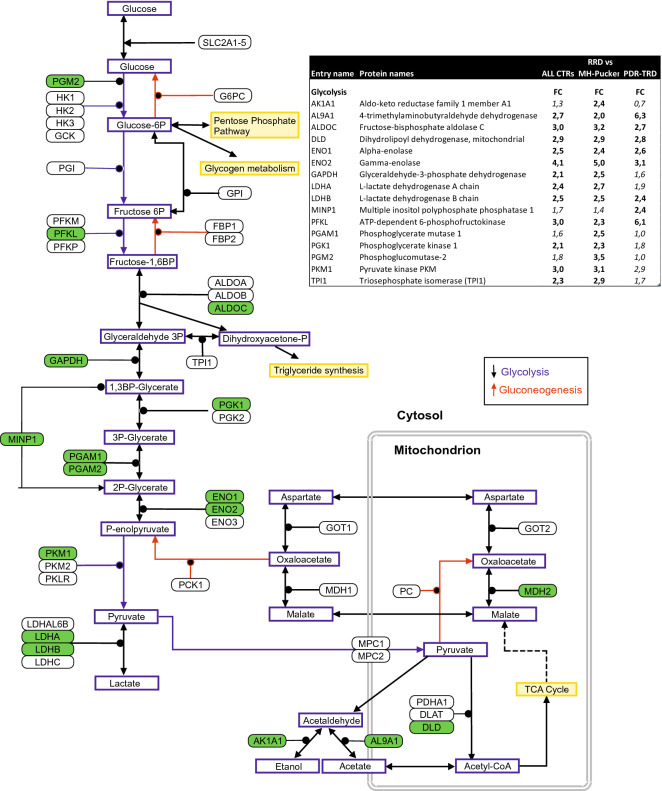
RRD enriches glycolysis enzymes in the vitreous. Proteomics analysis showed 21 enriched protein in RRD involved in glycolysis. These RRD up-regulated proteins in the glycolysis pathway are highlighted in green and listed in the table. Fold changes marked in bold in table are significant (q < 0.1). Figure modified from WikiPathways (http://www.wikipathways.org).

The second most enriched pathway was *‘*glycolysis’, a process of converting glucose into pyruvate and generating small amounts of ATP (energy) and NADH (reducing power). The RRD enriched glycolysis enzymes cover almost the entire pathway from glucose to lactate (Fig. 5). In addition, a clear reference to alternative energy production systems such as amino acid metabolism and citrate cycle was observed (KEGG pathways, Supplementary Table S4), which indicates the high energy requirement of the retina and strongly indicates that detachment of the retina is an energy consuming state.

Although inflammation events have been associated with RRD^21^, only a few inflammatory terms were observed over-represented in the RRD. One of the terms, ‘platelet degranulation’, contains several proteins involved in blood coagulation (Coagulation factor V, von Willebrand factor), complement activation (CLUS), or endopeptidase activity (TIMP1, AACT), suggesting that intraocular wound healing mechanism is active or activated in RRD. Additionally, several cell adhesion molecules, participating in wound healing and a scar formation, were upregulated in RRD samples compared to controls.

### Pathways involved in RRD

**Figure 6:**
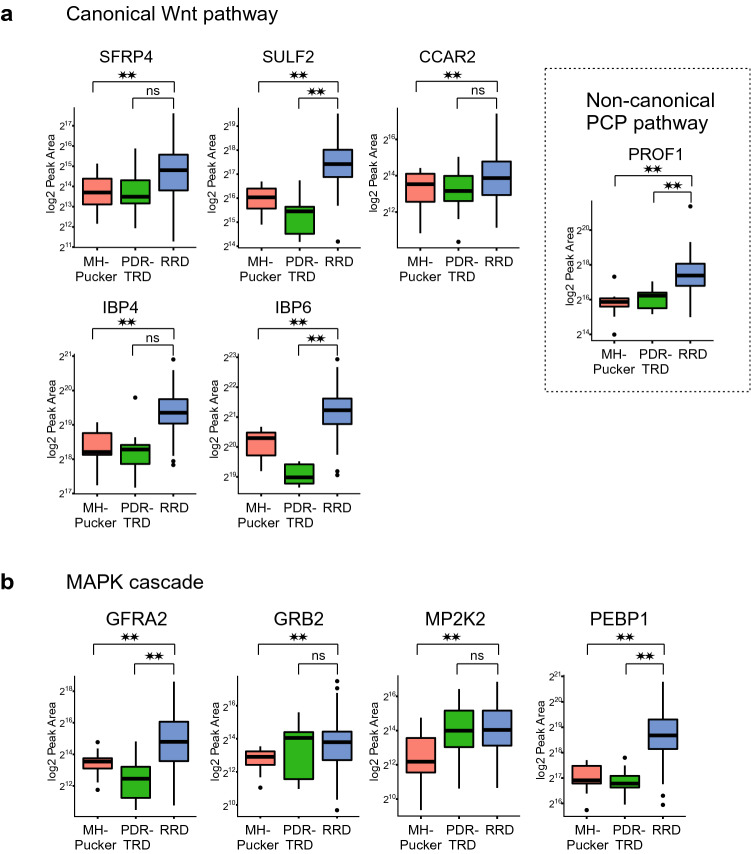
Signaling proteins upregulated in RRD samples. Boxplots of differentially expressed protein in EMT-related signal transduction cascades: in (**a**) Wnt-signaling and (**b**) MAPK cascade. *PCP *planar cell polarity pathway. log2 Peak Area indicates protein abundance in vitreous. **p < 0.1; *ns *non-significant differences.

Our data showed that two of the classical cellular signaling pathways, Wnt signaling and MAPK cascade, were enriched in RRD. A common RRD enriched factor for these two signaling pathways was the proteasome complex. The main function of ubiquitin–proteasome system in the eyes is the protein quality control, but it also has a role in signaling transduction by regulating protein stability^22^. However, the Wnt signaling and the MAPK cascade also have pathway specific RRD upregulated proteins (Fig. 6a). SFRP4 functions as modulators of Wnt signaling through direct interaction with Wnt ligand. SULF2 and CCAR2 work as positive regulators of the pathway, whereas, insulin-like growth factor-binding proteins (IBP4 and IBP6) are inhibitors of the canonical Wnt signaling and they provide a molecular cross-talk link between IGF signaling and Wnt signaling^23^. In addition, non-canonical Wnt/planar cell polarity (PCP) pathway regulates structure of the cytoskeleton through profilin 1 (PROF1) which was also detected in RRD proteome in high level.

The MAPK cascade is activated by a variety of extracellular stimuli factors or signal molecules, which trigger signal transduction by activating MAPKK kinase (MAPKKK), MAPK kinase (MAPKK), and MAPK, successively. The only MAPK family membrane kinase detected in our analysis was MAPK kinase 2 (MP2K2, also MEK2) which activates the ERK1 and ERK2 MAP kinases (Fig. 6b). However, additional upstream MAPK cascade regulators GFRA2, GRB2 and PEBP1 were observed (Fig. 6b). GFRA2 is a cell membrane receptor which mediates the activation of receptor tyrosine kinases (RTK). GRB2 is an adapter protein that provides a critical link between RTKs and the Ras signaling pathway which operates in MAPK cascade. And PEBP1 acts as a competitive inhibitor of MEK phosphorylation. Of note is that, MAPK cascade was significantly enriched only when RRD proteome was compared to MH-Pucker proteome and not when compared to PDR with TRD. This could suggest a more general importance of MAPK signaling for retinal detachment.

### Proteomic variations from a less severe form to more serious RRD

**Figure 7:**
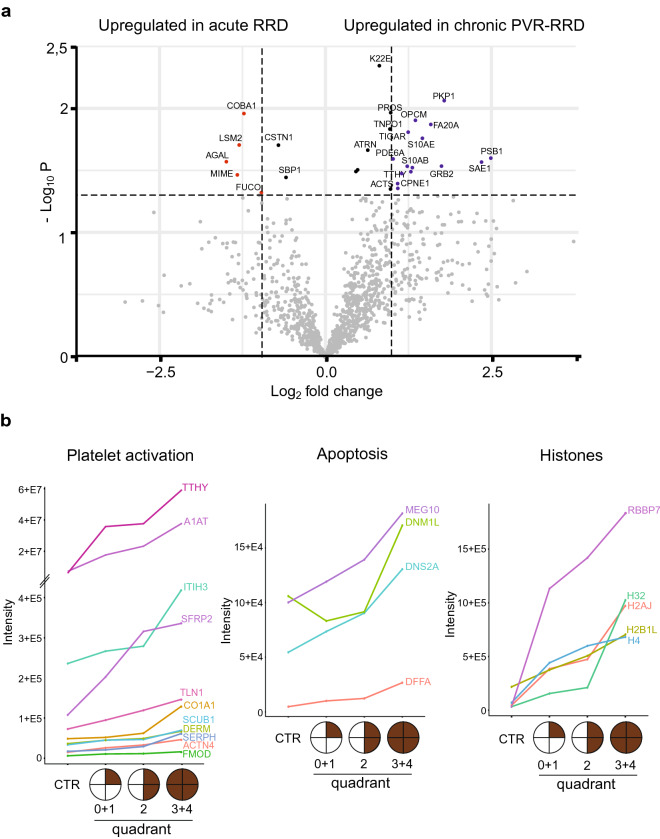
Protein abundances in acute RRD versus chronic RRD. (**a**) The volcano plots differentially expressed proteins between acute (area of retinal detachment less than one quadrant 0, n = 5) and chronic RRD (RRD with PVR, n = 8). The blue dots indicate significantly (p < 0.05) upregulated (FC < 2) proteins in chronic RRD and the red dots upregulated protein in acute RRD. Black dots indicate significantly differed proteins with FC < 2. (**b**) Progressive increase of protein abundance according to increasing RD quadrants. Proteins are classified according to whether they are associated with platelets activation (except TTHY), apoptosis or histones. Control samples (CTR) include MH, Pucker and PDR-TRD samples (n = 24). RRD patients with retinal detachment (RD) quadrant 0 and 1 were combined (n = 91), as well as quadrant 3 and 4 (n = 7). Mean protein intensity is displayed on log2-scale.

Proliferative vitreoretinopathy (PVR), the most common complication of RRD, comprises glial and RPE cells that migrate and generate membranes usually on the lower quadrants of retina. To understand the biological processes behind PVR development, we next determined the vitreous proteins which increased from acute RRD to chronic RRD. RRD forms with less than one quadrant retinal detachment were classified here as an acute onset RRD (RRD quadrant 0, n = 5), and RRD with PVR as a chronic RRD (n = 8). Altogether, 29 proteins were differentially detected in acute and chronic conditions (p < 0.05, Fig. 7a; Supplementary Table S6), of which 15 were upregulated in PVR (> 2-fold difference). These proteins were associated with GO-terms ‘cell adhesion’ (PKP1, OPCM, ANXA2, ACTN1), ‘apoptotic processes’ (S10AE, TIGAR) and ‘signaling’ (PSB1, GRB2, FA20A, S10AB, CPNE1, PDA6A). One of the most interesting upregulated proteins in the PVR eye was transthyretin (TTHY). TTHY is highly expressed RPE cells where it is serves as transporters of retinol to other cells^24^. In addition, chronic RRD with PVR samples were compared to PDR-TRD samples since both diseases represent an inflammatory and fibrotic conditions but PDR-TRD is always angiogenic and has a neovascularization process. Clear differences in proteomes can be observed between sample groups, as seen in PCA and hierarchical clustering analysis (Supplementary Fig. S2), further underlining the uniqueness of the RRD-PVR proteome.

Another way to study differences between the milder stage of RRD and more severe RRD is to analyze proteins whose abundance in vitreous increased according to increasing RD severeness e.g. quadrants. To get a sufficient number of samples in each group, we combined samples with quadrants 0 and 1, and samples with quadrants 3 and 4, to give a total of 3 groups; quadrant 0 + 1 (n = 91, less severe), quadrant 2 (n = 29) and quadrant 3 + 4 (n = 7, more severe RRD). We identified 66 proteins with increased presence in vitreous as RRD disease progressed (Supplementary Table S7). These identified proteins strongly suggest increased platelet activation during RRD since we detect many proteins involved in platelet degranulation (ITIH3, TLN1, ACTN4), blood coagulation (CO1A1, H32, A1AT, SCUB1) and collagen fibril organization (SFRP2, DERM, FMOD, CO1A1, SERPH) (Fig. 7b). In addition, various apoptotic proteins such as DNS2A, DFFA, DNM1L and MEG10, were upregulated during the RRD process. This, together with the observation of an increased amounts of histones (H2AJ, H2B1L, H4, H32) or histone-binding proteins (RBBP7, AN32E) in the vitreous, clearly indicates increased cell death as a result of extended retinal detachment. And importantly, the higher abundance of TTHY is also observed in this analysis, making it a potential biomarker of RRD.

## Discussion

As the incidence of RRD is increasing^25^ there is a dire need for diagnostics and efficient therapies for the RRD condition. Better understanding of the possible endogenous molecular triggers for RRD enables new therapeutic and diagnostic tools. In the present study, we performed quantitative in-depth molecular pathology analysis of the vitreous proteomes of 127 RRD patients. We identified a total of 1 177 human proteins in our analysis, out of which more than 60% were annotated to “extracellular exosomes'' in the cellular component analysis. Exosomes are small functional biovesicles, actively released into vitreous from the neighboring cells, transporting complex components to target tissues, and participating in intercellular communication such as the immune response, signal transduction or cell-to-cell communication^26^. A recent publication showed that exosomes are the major part of the vitreous in which they are thought to participate in the dynamic interaction between the vitreous and the retina^27^. Exosomes could also indicate a unique mechanism for the release of intracellular components into the vitreous in RRD. However, we cannot rule out the possibility that structural changes or cell death occur in the RRD condition, resulting in the release of intracellular components into the extracellular space.

One of the main findings was a high number of photoreceptor proteins in the RRD vitreous compared to other ophthalmic diseases. Photoreceptors are non-dividing and terminally differentiated neurons with a very specific role in the first stage of vision. The earliest structural effects of RRD can be seen on the outer segments (OS) of the photoreceptors and RPE cells. These two structures are connected through the numerous microvilli present on the apical surface of the RPE through which RPE provides the major metabolic and nutritional support for the photoreceptors. In RRD pathology, loss of oxygen or glucose transport along the connecting cilia leads to caspase-associated photoreceptor cell death and permanent visual loss^28^. The accumulation of these specialized photoreceptor cell proteins in RRD proteome confirms a large photoreceptor cell death after RRD. Therefore, effective neuroprotective approaches are needed and inhibition of photoreceptor cell death and apoptosis via drug therapy could drastically improve the final visual prognosis of RRD patients.

Another interesting finding was the high number of proteins related to carbon metabolism and glycolysis. The visual system is one of the highest energy demanding tissue in the human body, and the photoreceptors are the most metabolically active cells in the retina^29^. Previous studies have shown that the mammalian retinal function is dependent on glycolysis^30^. Indications of increased energy metabolism during RRD have been shown previously^17^, and they suggested that retinal cells try to compensate for metabolic stress by increasing energy production after RRD. However, the consequence of this is, that these glycolytic proteins are released into vitreous as the disease progresses to a more difficult condition.

During the RRD process and especially during the early repair phase, phagocytic cells in immune response such as neutrophils, monocytes, and macrophages can migrate to the damaged subretinal space along with migrating RPE cells^7^. Monocytes are the primary immune cells mediating the cytokine storm following RRD. However, only a few cytokines were detected in our analysis because these molecules are present in vitreous at very low undetectable levels. After the acute phase, neutrophils take care of phagocytosis and debridement (proteolytic enzymes). Platelets participate in immune reactions, and express proteasomes and MHC class 1 molecules^31^. The coagulation cascade is activated, accumulation of fibrin leads to fibrosis, and initial regenerative process leads to mature scar tissue (PVR). Several proteins involved in platelet degranulation mechanism were detected at high level in RRD samples in our analysis. The amount of these proteins increased as RRD progressed, suggesting an important role for platelets activation in the RRD process.

Of the main cellular signaling pathways, Wnt and MAPK cascades were the most affected signaling pathways in our analysis. Wnt signaling modulates cellular and tissue differentiation, and it has been shown to have an essential role in eye development, especially in retinal vascular morphogenesis^32^. Wnt signaling has also been linked to several vascular eye diseases, like wet age-related macular degeneration or DR^33^. Importantly, epithelial mesenchymal transition-related signal transduction cascades, including Wnt/beta-catenin signaling, are known to be activated in RRD eyes^34^. The mitogen-activated protein kinases (MAPKs) are important mediators of signal transduction and play a key role in development and inflammatory responses. In the retina, MAPKs regulate apoptosis during retinal development^35^. The activation of the MAPK cascade has been shown to be especially important in Müller glial cells formation during retina development^36^ and retinal detachment^37,38^, MAPK cascade has also been suggested to have indirect neuroprotective action for the photoreceptors in RD^39^. Interestingly, many of the identified signaling proteins were associated with proteasome-mediated protein degradation. The ubiquitin–proteasome system (UPS) has a key role in keeping up cellular protein homeostasis by its ability to degrade non-functional self-, foreign, or short-lived regulatory proteins^40^. Increasing evidence suggests that UPS dysfunction is a major pathogenic mechanism in ophthalmic degenerative disorders^41^.

The pathological mechanisms of RRD in the acute phase differ from the chronic phase characterized by PVR. By comparing acute RRD to chronic with PVR, we obtained evidence that proteins involved in cell adhesion, apoptotic processes, and wound processes would affect RRD progression or PVR formation. This is in line with a previous report showing that the extent of RRD significantly influences intravitreal proinflammatory, profibrotic, and proapoptotic protein expression^42^. Increased intravitreal expression of soluble apoptosis and adhesion molecules at the time of primary RRD surgery is also known to be associated with future development of PVR^43^. Our data reveals that several important alterations are present in RRD vitreous also in protein level and highlight apoptotic and fibrotic components as potential therapeutic targets to prevent RRD process and conversion of RRD into the most severe form. In conclusion, preventing harmful PVR processes in RRD eyes is of utmost importance. Deeper understanding of the complex wound-healing mechanisms would help to develop effective therapeutic immunomodulatory medication.

In a recent paper, Lauwen et al. listed several suggested biomarkers in ophthalmology recovered by high-throughput methods, including genomics, transcriptomics, and metabolomics^44^. Genome-wide association study (GWAS) point out the potential role for cell adhesion and migration and apoptosis of photoreceptors and RPE cells in the pathology of RRD^45^. We can further support these findings, because we find strong indicators of photoreceptor apoptosis in our data. Also several inflammatory response factors were listed as potential biomarkers; however, we found no evidence of this in our data, although many immune response proteins are present in our data. This might suggest a larger role of inflammatory response in PDR-TRD, Pucker and MH than in RRD. In addition, Johnston et al. listed several genes associated with syndromic and non-syndromic forms of RRD^46^. Some of these proteins that were indicated on genome level as potential risk factors, were also detected in our proteomic data. In particular, collagen or collagen related proteins were more abundant in RRD sample groups (especially TGFBI, TIMP1, COL11A1). Also MMP2 and COL6A1 differ significantly between RRD and MH-Pucker control group. This indicates a distorted architecture of vitreous collagen fibrils in RRD eyes that acts with an altered force at the vitreous-retinal interface which can expose to a posterior vitreous detachment or retinal tear.

In our analysis, three previously reported biomarkers (B2M, CLUS, and TTHY) had a statistically significant difference between RRD and control samples^44^. TTHY is synthesized and secreted by RPE cells, contributing to the formation of fibrous membranes on the retina in PVR-RRD eyes. Previously, Shitama et al. suggested that TTHY could be a candidate biomarker of fibrosis in RRD eyes^47^. Because TTHY was observed at a very high level in RRD vitreous and we could see a clear increase in protein abundance during RRD progression, we believe that increased TTHY levels in RRD patients prognosticates towards PVR. In addition, histones or histone-binding proteins were found to be RRD specific molecules. Histones can be released extracellularly by oxidative stress and after tissue injury^48^. Because histone levels were very low in control samples, and increased significantly during RRD, these molecules could be potential candidates for RRD biomarkers.

Despite numerous efforts, there are no effective preventive or pharmacological treatment strategies to reduce the risk of PVR fibrosis development in eyes with RRD^10,11^. Historically to prevent fibrosis, many compounds, known to modify wound healing, have been studied including anti-inflammatory agents (low molecular weight heparin, steroids, statins) as well as cytotoxic and immunosuppressive drugs (such as 5-fluorouracil, daunorubicin, methotrexate)^49,50^. More recently, Fucoidan was shown to reverse TGF beta1-induced EMT of RPE cells, and suppress formation of alpha-SMA and fibrosis^51^. We are actively looking for new target molecules and biomarkers for RRD so that one day we can offer more individualized treatments to patients suffering from this potentially blinding eye disease.

In summary, our data provides the most detailed insight into the dramatic proteome changes that occur in vitreous humor during RRD. Specifically, the proteins involved in phototransduction and glycolysis were enriched as well as signaling molecules from Wnt and MAPK pathways. Retinal regeneration without fibrosis in RRD pathology remains a challenge, and we believe that our study can help to shed light on the molecular mechanisms active prior, during and after the RRD. Understanding the molecules and the mechanisms involved (in time and space) is the necessity to develop new therapies for RRD patients.

## Methods

### Patients

The study was conducted according to the tenets of the Declaration of Helsinki and approved by the Institutional Review Board of Helsinki University Central Hospital at the University of Helsinki in Finland. Signed informed consent was obtained from each participant before the sampling occurred. Confidentiality of the patient records was maintained when the clinical data were entered into a computer-based standardized data entry for analysis.

Patients in our study were admitted for primary vitrectomy due to RRD (n = 127). Patients originate from two separate cohorts, the first set (n = 72, collected during 2006–2008), and the second set (n = 55, collected during 2010–2017). Proliferative vitreoretinopathy (PVR) in RRD eyes was graded according to the classification of the Retina Society Terminology Committee (1991)^52^. The findings were compared with three previously studied ophthalmic diseases, macular hole (MH; n = 5), Pucker (n = 10) or proliferative diabetic retinopathy (PDR) eyes with tractional retinal detachment (TRD; n = 9)^15,16^. Eye examination included measurement of visual acuity, intraocular pressure, axial length, and biomicroscopy of anterior and posterior segment of the eye.

### An optical coherence tomography

The optical coherence tomography (OCT) scan was obtained with Heidelberg Eye Explorer (Version 1.10.2.0, Heidelberg Engineering GmbH, 2017).

### Vitreous collection and sample preparation

All vitrectomies were performed by the recruiting vitreoretinal surgeon. Undiluted vitreous samples (up to 1000 μl) were collected at the start of the conventional 3‐port pars plana vitrectomy (Alcon Instruments, Inc. or Alcon Constellation Vision system) without an infusion of artificial fluid. The samples were immediately frozen and stored at − 70 °C. Total protein content of the samples was measured using a BCA protein assay kit (Pierce, Thermo Scientific).

### Vitreous reference spectral library generation

Four samples, covering the diseases RRD, MH, Pucker and PDR, were selected for the specific vitreous ion library generation. 500 µg of total protein were reduced with TCEP (Tris(2-carboxyethyl)phosphine; Sigma Aldrich), alkylated with iodoacetamide, trypsin-digested with Sequencing Grade Modified Trypsin (Promega) using a 1:100 enzyme:protein ratio at 37 °C o/n, and then desalted with C18 microspin columns (Nest Group). The formed tryptic peptides were fractionated with an Agilent 3100 OFFGEL fractionator (Agilent Technologies), using 12 cm pH 3–10 IPG strips (GE Healthcare). The strips were focused at a maximum of 8000 V, 50 µA, 200 mW, until 50 kVhrs was reached. The runs took approximately 24 h. The four adjacent fractions were combined to give a total of six fractions per samples, which were desalted with C18 columns and analyzed separately with a TripleTOF 6600 Quadrupole Time-Of-Flight (Sciex) and Orbitrap Elite EDT Hybrid MS (Thermo Scientific). TripleTOF 6600 was coupled to an Eksigent nanoLC with a Turbo V Source, working in microspray mode, and YMC-Triart C18 column (12 nm, 3 µm, 150 × 0.3 mm) was used for peptide separation. Orbitrap Elite was coupled with an EASY-nLC II system via a nanoelectrospray ion source, and Acclaim PepMap 100 column (75 μm × 15 cm, 2 μm, 100 Å) was used. The MS analyses were performed in data-dependent acquisition (DDA) in positive ion mode, using linear 60 min gradient.

Raw data were processed with ProteinPilot software using Paragon algorithm (v4.5, AB SCIEX)^53^. The original raw-files from Orbitrap Elite were converted to mgf files using the conversion tool MSConvert. Wiff-files from TripleTOF were used as such. Searches were done against the human UniProtKB database (release 11/2018, The UniProt Consortium 2019) supplemented with porcine trypsin and iRT-peptide sequences (total of 20,347 entries). The search parameters were as follows: sample type, identification; Cys alkylation, iodoacetamide; digestion, trypsin; special factors, none. The “Thorough ID” mode was selected, which automatically adjusts the mass tolerance to fit the high‐resolution MS and MS/MS data. The results were filtered to a maximum false discovery rate (FDR) of 5%, resulting in the human vitreous humor spectral library containing 272,218 spectra from 18,446 distinct peptides which can be used to quantify 1558 proteins.

### SWATH analysis and peak extraction

For SWATH-analysis, 100 µg of total protein per sample was digested as described above. SWATH-MS analysis was performed using TripleTOF 6600. Precursor ion selection was done in the 400–1250 m/z range, with a variable window width strategy (from 6 to 50 Da). Peptide activation was performed using CID, using nitrogen as inert gas, with rolling collision energy, and 5 eV of energy spread. The accumulation time was set to 250 ms for MS1 and 100 ms for MS2 scan. The entire duty cycle was approximately 3.1 s.

Peak extraction of the SWATH data was performed using PeakView (version 2.1) with SWATH quantitation plug-in (SCIEX). Reference peptides from the iRT-kit (Biognosys) spiked into each sample were used to calibrate the retention time of extracted peptide peaks. The settings used were as follows: the maximum number of peptides per protein, 25; the number of transitions or fragment ions per peptide, 6; a peptide confidence threshold, 90; a False Discovery Rate (FDR), 1%; XIC (Extracted Ion Chromatogram) retention time window, 10; m/z tolerance, 75 ppm; shared and modified peptides were excluded. After SWATH peak extraction, the transition ion peak areas, peptide peak areas, and protein peak areas were exported in Excel format for further statistical analysis.

### Statistical analysis

The protein peak areas were normalized by total area normalization, i.e. average total intensity of all samples was divided by the total area of each sample. The data was further analyzed with RStudio version 1.2. running with R version 3.6.1. To discover proteins that were significantly altered between RRD-samples and controls Welch’s t-test was used. Welch’s t-tests p-values were multiple testing corrected using the FDR method and differences with q-value < 0.1 and greater than twofold change in intensity were considered significant. Principal component analysis (PCA) and Ward hierarchical clustering were done on log-scaled and z-normalized data. Statistical analysis was done using an in-house R script. Ward Hierarchical Clustering was performed using the R function hclust with the Euclidean distance metric.

### Functional annotations

Gene Ontology (GO) annotations and KEGG pathways were obtained from DAVID bioinformatics resources (https://david.ncifcrf.gov/)^54,55^.

## Supplementary Information

Supplementary Tables.

Supplementary Figures.

## Data Availability

Raw mass spectrometry data have been deposited to the MassIVE database with ID: MSV000086412 (https://massive.ucsd.edu).
