# Supplementary material for: Molecular pathogenesis of rhegmatogenous retinal detachment
Source: Sci Rep. 2021 Jan 13;11:966. doi: 10.1038/s41598-020-80005-w (PMC7806834; doi:10.1038/s41598-020-80005-w)
Supplement: Supplementary file 2 — Supplementary Figures. [file 41598_2020_80005_MOESM2_ESM.pdf]

Supplementary Information

Molecular pathogenesis of rhegmatogenous retinal detachment

Tiina Öhman [1], Lisa Gawriyski [1], Sini Miettinen [1], Markku Varjosalo [1,3,*], Sirpa Loukovaara [2,3,*]

[1]Institute of Biotechnology and Helsinki Institute of Life Science, University of Helsinki, Viikinkaari 1, P.O. Box 65, FI-00014 Helsinki, Finland

[2]University of Helsinki and Unit of Vitreoretinal Surgery, Department of Ophthalmology, University of Helsinki and Helsinki University Hospital, Haartmaninkatu 4 C, FI-00290 Helsinki, Finland

[3] These authors contributed equally as senior authors.

***Corresponding author:**

Markku Varjosalo, PhD, Institute of Biotechnology and Helsinki Institute of Life Science, University of Helsinki, Finland. E-mail: markku.varjosalo@helsinki.fi

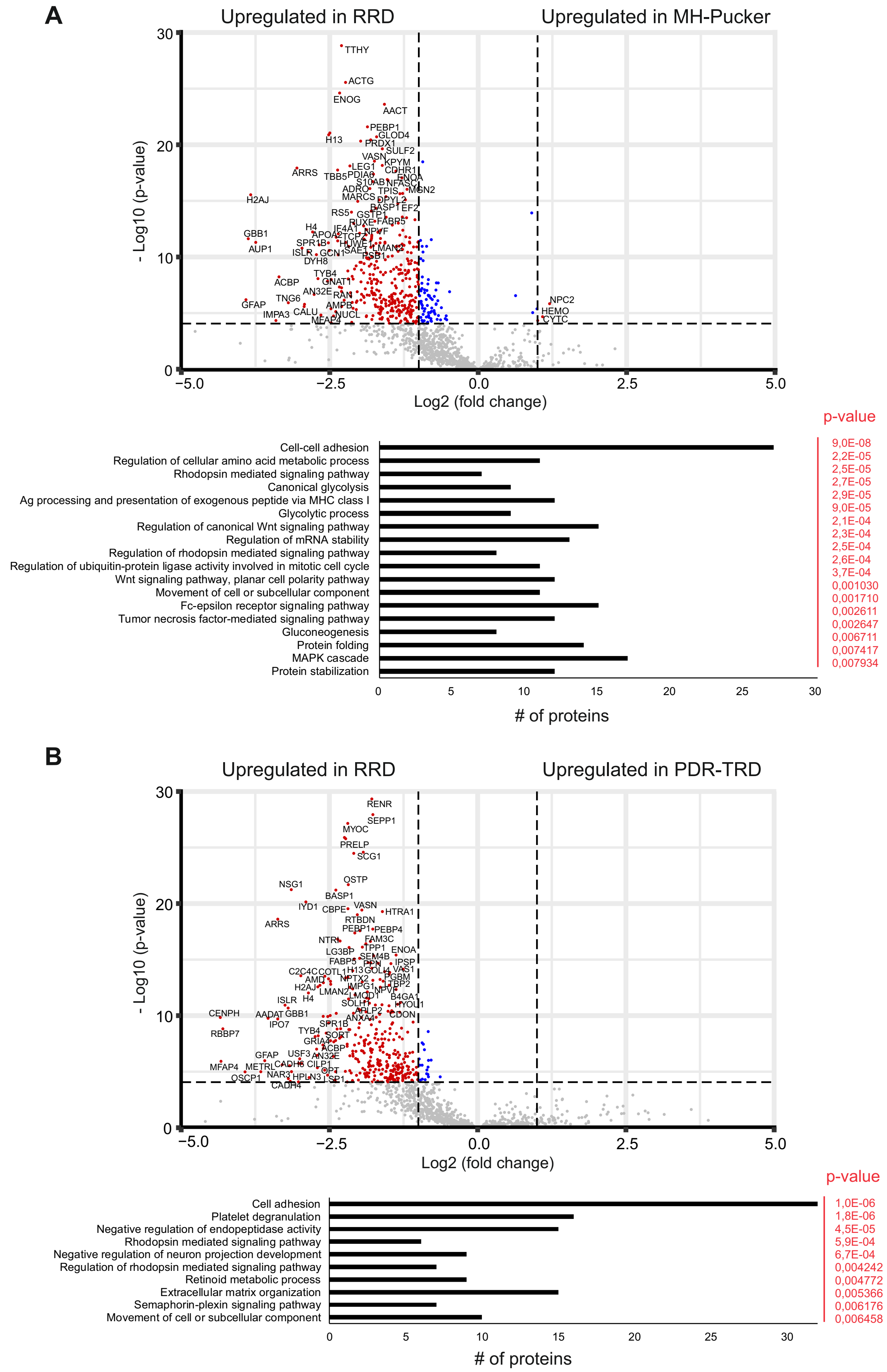

Figure S1:**Comparison between RRD and control posterior segment eye disease proteomes**. A) Differences between RRD and MH-Pucker and B) between RRD and PDR-TRD proteomes. The volcano plots of differentially expressed proteins between RRD and control eye groups. P-value was set to match q-value < 0.1. The red dots indicate significantly differed proteins with q < 0.1 and FC > 2. Significantly upregulated proteins in the RRD samples were categorized by their biological processes in both comparisons (A+B).

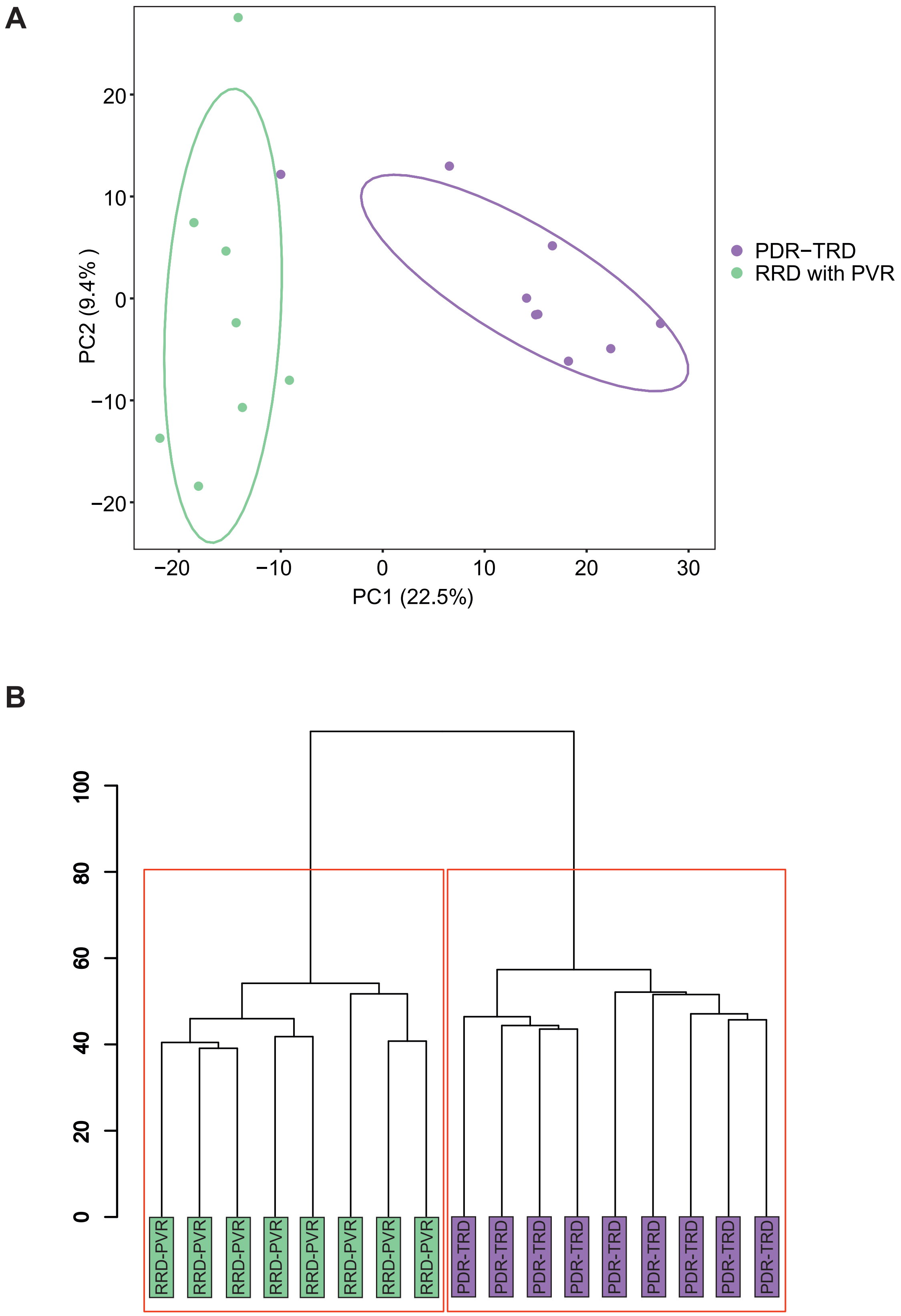

Figure S2: **Comparison between RRD-PVR and PDR-TRD proteomes.** A) PCA analysis demonstrated a clear separation between RRD with PVR and PDR-TRD sample groups. B) A similar result was obtained using hierarchical clustering, in which RRD-PVR samples clustered separately from PDR-TRD samples.
